# Complete plastid genome sequence of a medical herb, *Anisomeles indica* (L.) Kuntze (Lamiaceae)

**DOI:** 10.1080/23802359.2019.1659112

**Published:** 2019-08-27

**Authors:** Gang Yao, Jiuxiang Huang, Yuling Li

**Affiliations:** Guangdong Key Laboratory for Innovative Development and Utilization of Forest Plant Germplasm, College of Forestry and Landscape Architecture, South China Agricultural University, Guangzhou, China

**Keywords:** Anisomeles indica, Lamiaceae, plastid genome, phylogenomics

## Abstract

The species *Anisomeles indica* is used medicinally and widely distributed from India, to China and the Indo-China Peninsula. The first complete plastid genome sequence of *A. indica* reported here was 151,900 bp long, with the large single copy (LSC) region of 83,144 bp, the small single copy (SSC) region of 17,556 bp and two inverted repeats (IRa and IRb) of 25,600 bp. The plastome contained 113 genes, including 79 protein-coding genes, 4 ribosomla RNA genes and 30 transfer RNA genes. The overall GC content was 38.26%. Result from phylogenetic analysis suggests that *Anisomeles* is closely related to the genus *Pogostemon*.

*Anisomeles* R. Brown belongs to the tribe Pogostemoneae, subfamily Lamioideae, family Lamiaceae (Li et al. 2016; Yao et al. 2016). It consists of about five or six species of herbs (Li and Hedge 1994), among which the species *Anisomeles indica* (L.) Kuntze is a strong-scented herb, occurring widely from India to China and the Indo-China Peninsula, and it is found usually growing along forest margins, roadsides and waste areas (Li and Hedge 1994; Batish et al. 2012). The species is used medicinally for rheumatism, colds, fevers, liver disease and protection, and snake bites (Li and Hedge 1994; Rao et al. 2009).

Samples of *Anisomeles indica* were collected from Guangdong province (China; N23°11′7″, E113°21′53″). Voucher specimen (*G. Yao 448*) was deposited in the Herbarium of South China Botanical Garden, Chinese Academy of Sciences (IBSC). Total DNA was isolated using the CTAB method (Doyle and Doyle 1987) and sequenced on the Illumina HisSeq 2500 Sequencing System. Reads were assembled with the pipeline GetOrganelle (Jin et al. 2018), and the genome obtained was annotated using software PGA (Qu et al. 2019). The annotated plastid genome sequence has been deposited into the GenBank with the accession number MN115836.

The whole plastid genome of *Anisomeles indica* was 151,900 bp in length, with a large single-copy (LSC) region (83,144 bp), a small single-copy (SSC) region (17,556 bp), and a pair of inverted repeats (IRa and IRb; 25,600 bp). The annotated genome comprised 113 genes, including 79 protein-coding genes, four ribosomal RNA genes (rrn 16, rrn23, rrn4.5, rrn5), and 30 transfer RNA genes. Seventeen genes were duplicated in the IR regions, including six protein-coding genes (*ndhB*, *rpl2*, *rpl23*, *rps12*, *rps7*, *ycf2*), four ribosomal RNA genes (rrn16, rrn23, rrn4.5, rrn5), and seven transfer RNA genes (trnA-UGC, trnI-CAU, trnI-GAU, trnL-CAA, trnN-GUU, trnR-ACG, trnV-GAC). The overall GC content of *A. indica* plastid genome is 38.26% (LSC, 36.38%; SSC, 32.29%; IRs, 43.37%).

The maximum likelihood (ML) phylogenetic tree was constructed using RAxML-HPC2 (8.1.24) (Stamatakis 2014) on the CIPRES cluster (Miller et al. 2010), employing the GTR + G model and the default number of rate categories (C = 25). We conducted as rapid bootstrap analysis using the GTR + G model with 1000 bootstrap replicates. Phylogenetic analysis based on 79 protein-coding genes of 24 representative plastomes within the family Lamiaceae suggests that *Anisomele*s is closely related to the genus *Pogostemon* (Figure 1).

**Figure 1.**
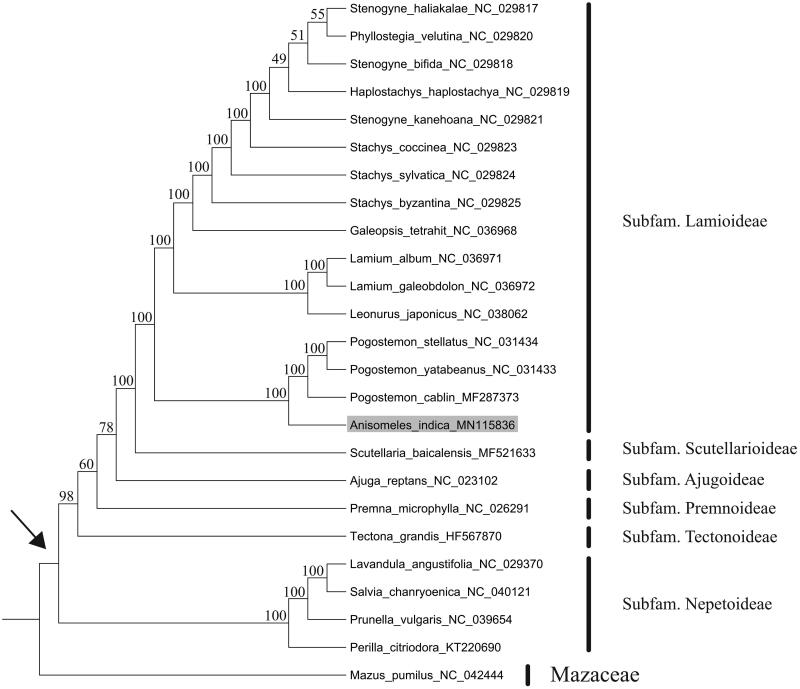
Maximum likelihood tree of Lamiaceae inferred from 79 protein-coding genes of 25 plastomes (including the outgroup *Mazus pumilus* from Mazaceae). Bootstrap values are indicated above branches. The crown node of Lamiaceae is shown by an arrowhead. The species *Anisomeles indica* is marked with gray background.

## References

[CIT0001] Batish DR, Singh HP, Kaur M, Kohli RK, Singh S 2012 Chemical characterization and phytotoxicity of volatile essential oil from leaves of *Anisomeles indica* (Lamiaceae). Biochem Syst Ecol. 41:104–109.

[CIT0002] Doyle JJ, Doyle J 1987 A rapid DNA isolation procedure for small quantities of fresh leaf tissue. Phytochem Bull. 19:11–15.

[CIT0003] Jin JJ, Yu WB, Yang JB, Song Y, Yi TS, Li DZ 2018 GetOrganelle: a simple and fast pipeline for de novo assembly of a complete circular chloroplast genome using genome skimming data. BioRxiv. doi:10.1101/256479

[CIT0004] Li B, Cantino PD, Olmstead RG, Bramley GLC, Xiang CL, Ma ZH, Tan YH, Zhang DX 2016 A large-scale chloroplast phylogeny of the Lamiaceae sheds new light on its subfamilial classification. Sci Rep. 6(1):34343. 27748362 10.1038/srep34343 PMC5066227

[CIT0005] Li HW, Hedge IC 1994 Lamiaceae In: Wu Z.Y. and Raven P.H (Eds.) Flora of China 17. St. Louis (MO): Science Press, Beijing & Missouri Botanical Garden Press, pp. 50–299.

[CIT0006] Miller MA, Pfeiffer W, Schwartz T 2010 Creating the CIPRES Science Gateway forinference of large phylogenetics trees In: Proceedings of the Gateway Computing Environments Workshop (GCE). New Orleans, LA, pp. 1–8.

[CIT0007] Qu XJ, Moore MJ, Li DZ, Yi TS 2019 PGA: a software package for rapid, accurate, and flexible batch annotation of plastomes. Plant Methods. 15:50. 31139240 10.1186/s13007-019-0435-7 PMC6528300

[CIT0008] Rao YK, Fang SH, Hsieh SC, Yeh TH, Tzeng YM 2009 The constituents of *Anisomeles indic*a and their anti-inflammatory activities. J Ethnopharmacol. 121(2):292–296. 19041702 10.1016/j.jep.2008.10.032

[CIT0009] Stamatakis A 2014 RAxML version 8: a tool for phylogenetic analysis and post-analysis of large phylogenies. Bioinformatics. 30(9):1312–1313. 24451623 10.1093/bioinformatics/btu033 PMC3998144

[CIT0010] Yao G, Drew BT, Yi TS, Yan HF, Yuan YM, Ge XJ 2016 Phylogenetic relationships, character evolution and biogeographic diversification of *Pogostemon* s.l. (Lamiaceae). Mol Phylogenet Evol. 98:184–200. 26923493 10.1016/j.ympev.2016.01.020

